# The duration of daily activities has no impact on measures of overall wellbeing

**DOI:** 10.1038/s41598-021-04606-9

**Published:** 2022-01-11

**Authors:** Amanda Henwood, João Guerreiro, Aleksandar Matic, Paul Dolan

**Affiliations:** 1 The London School of Economics and Political Science, London, UK; 2 Koa Health, Boston, USA

**Keywords:** Physiology, Environmental social sciences

## Abstract

It is widely assumed that the longer we spend in happier activities the happier we will be. In an intensive study of momentary happiness, we show that, in fact, longer time spent in happier activities does not lead to higher levels of reported happiness overall. This finding is replicated with different samples (student and diverse, multi-national panel), measures and methods of analysis. We explore different explanations for this seemingly paradoxical finding, providing fresh insight into the factors that do and do not affect the relationship between how happy we report feeling as a function of how long it lasts. This work calls into question the assumption that spending more time doing what we like will show up in making us happier, presenting a fundamental challenge to the validity of current tools used to measure happiness.

## Introduction

It is a substantive fact that feeling happy for longer will make you happier overall. Many behavioural interventions designed to bolster wellbeing focus on increasing the time we spend in pleasurable moments, since it is arguably far easier to control our allocation of time that it is our emotional states. Given the importance of time for happiness, measuring the duration associated with our emotional states has long been considered a staple of happiness measurement. Therefore, the tools we use to measure happiness ought to show a meaningful impact of time on people’s happiness reports. But what if current tools used to measure happiness fail to demonstrate this?

Happiness, otherwise known as subjective wellbeing (SWB), refers to how people think and feel about their lives and their everyday experiences^1^. It is typically measured by capturing two major components: how happy we feel (intensity) and how long we feel it for (duration). Ecological Momentary Assessment (EMA) and The Day Reconstruction Method (DRM) are two of the most used methods for measuring these components^2,3^. EMA requires people to report on their happiness at specific moments in time throughout the day (usually selected randomly) alongside the activities that they are engaged in^2^. To capture the duration of each happiness episode using EMAs, participants must indicate how long the activity they are currently engaged in has lasted to that point. Instead of relying on “in the moment” reports, DRMs gather wellbeing reports from one point in time relating to a series of episodes that had occurred the previous day^4^. These wellbeing reports follow a diary-like format whereby people report episodes from the day (e.g. working) and how long they last until the entire day is covered.

Changes in SWB have been shown to predict important changes in health-related behaviours (e.g. sleep^5^). They are also increasingly being used to assess societal progress on a national scale^6,7^ and to evaluate policies and programmes^8^. Understanding the contribution of various factors, such as duration, to SWB is therefore crucial for advancing both science and society, and a subject of wide appeal to practitioners, clinical and health psychologists, policy makers, economists, and individuals^9–11^.

Evidence has already shown that the proportion of time spent in certain activities can impact overall SWB. For example, the proportion of time spent in happy relative to less happy activities has been shown to be an important determinant of overall SWB^12^. There is, however, currently a dearth of empirical research exploring whether current SWB measures are picking up on one of the most fundamental assumptions: namely, that more time spent in happy activities is better for us than spending less time in happy activities. SWB measures must meet this assumption if they are to reliably inform behavioural interventions targeting happiness.

## The present empirical research

To assess the impact of duration on reported happiness, we gathered daily EMA and DRM happiness reports using a mobile app over a 2–3-week period in two large, and diverse samples: one mixed (people of different ages, gender and nationality) and one student sample. Since SWB is determined by the purpose (or worthwhileness) of an activity as well as by its pleasure (or happiness), participants were asked to report both the worthwhileness and happiness in both EMA and DRM^13^. The required sample size to detect a medium effect size (0.50) with a high power criterion (90%) and standard significance level (α = 0.05) when comparing duration with non-duration weighted SWB is 43^14^. However, since we planned on exploring other variables as moderators, and to aid replicability of our results with an extended set of registered hypotheses^15^, we aimed to recruit as many participants as possible. Sensitivity power analysis with the final number of participants (217 for the student sample and 195 for the mixed sample) included in our analyses for the core hypothesis (ref section to the supplementary materials), suggested that our study had the power of 90% (α = 0.05) to detect Cohen’s d of 0.22 and 0.20 respectively, which is close to the small effect of 0.20.

### Ethics statement

All studies were conducted in line with GDPR and the guidelines of the American Psychological Association. All participants gave informed consent on joining the study and the experimental protocol was approved by the London School of Economics Ethics Committee. The study did not involve deception and hypotheses, methods, and analysis for the second of the two studies were pre registered. We applied many different robustness checks to ensure robustness of our findings, which can be found in the Supplementary Materials.

## Results

In analysing these data, we first report descriptive statistics of SWB intensity scores for each sample, method, and measure (see Table 1). These are presented in the table below.

**Table 1:** Descriptive statistics of SWB intensity scores for each sample, method, and measure, with confidence interval at 95% level.

Mean intensity	Student sample		Mixed sample	
	EMA	DRM	EMA	DRM
Happiness (CI@95%)	7.17 ± 1.83 [7.14, 7.20]	7.17 ± 1.76 [7.14, 7.21]	6.44 ± 1.91 [6.40, 6.48]	6.53 ± 1.98 [6.49, 6.58]
Worthwhileness (CI@95%)	7.34 ± 1.90 [7.30, 7.37]	7.42 ± 1.77 [7.39, 7.46]	6.39 ± 2.19 [6.34, 6.43]	6.57 ± 2.16 [6.52, 6.63]

We note that mean happiness intensity scores were slightly lower for EMA than for DRM in the student sample. The average reported happiness intensity for EMA and DRM in the student sample was (*EMA:* M = 6.44, SD = 1.91, CI (95%) = [6.40, 6.48]; *DRM:* M = 6.53, SD = 1.98, CI (95%) = [6.49, 6.58]). This difference was not present in the mixed sample. The average reported happiness intensity for EMA and DRM in the mixed sample was (*EMA:* M = 7.17, SD = 1.83, CI (95%) = [7.14, 7.20]; *DRM:* M = 7.17, SD = 1.76, CI (95%) = [7.14, 7.21]). Mean worthwhileness intensity scores were also slightly lower for EMA than for DRM in both samples. The average reported worthwhileness intensity for EMA and DRM in the student sample was (*EMA:* Mean = 6.39, SD = 2.19, CI (95%) = [6.34, 6.43]; *DRM:* Mean = 6.57, SD = 2.16, CI (95%) = [6.52, 6.63]). The average reported worthwhileness intensity for EMA and DRM in the student sample was (*EMA:* Mean = 7.34, SD = 1.90, CI (95%) = [7.30, 7.37]; *DRM:* Mean = 7.42, SD = 1.77, CI (95%) = [7.39, 7.46]). Overall, the mixed sample reported higher happiness and worthwhileness intensity than the student sample.

In terms of duration, the average reports for EMA and DRM in the student sample were (*EMA:* Mean = 154 min, Median = 120 min, SD = 118 min, CI (95%) = [152, 156]; *DRM:* Mean = 163 min, Median = 120 min, SD = 135 min, CI (95%) = [160, 166]). The average reported duration for EMA and DRM in the mixed sample was (*EMA:* Mean = 158 min, Median = 100 min, SD = 143 min, CI (95%) = [156, 160]; *DRM:* Mean = 168 min, Median = 120 min, SD = 151 min, CI (95%) = [165, 170]). Note that we doubled the EMA duration times to account for the fact that people are interrupted mid episode and enable comparison between measures. Taking these doubled EMA reports into account, DRM duration reports are about 10 min longer than EMA duration reports in both samples (statistically significant with *p* value < 0.001). Although significant, this is a small difference and likely due to differences in the recall style of each reporting method: EMAs rely on in the moment recollection whereas DRMs rely on recollection of the previous day. Further exploration of this difference is beyond the scope of the current paper.

**Figure 1:**
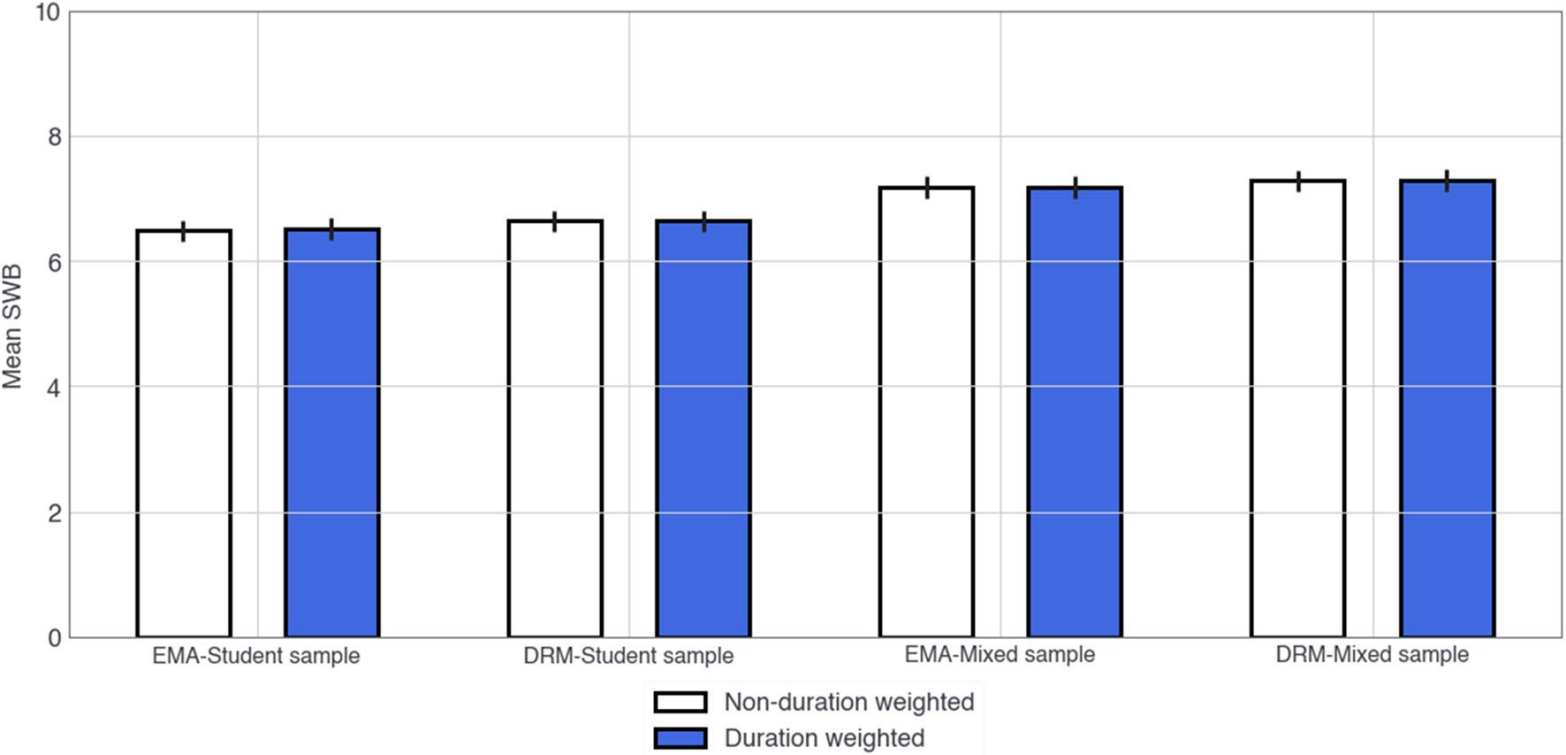
Mean SWB (happiness intensity × duration) scores with and without duration weights, for each questionnaire type and sample.

**Figure 2:**
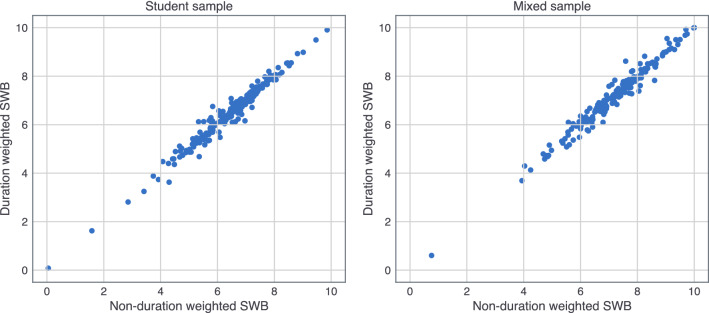
Scatter plots showing total reported SWB (happiness intensity × duration) scores per individual, with and without duration weights, reported in the EMA questionnaire. The figure on the left represents the student sample and the figure on the right represents the mixed sample.

The results speaking to the main research question—*how does duration-weighting contribute to overall SWB?* are presented in Figs. 1 and 2. For brevity, we report only results for happiness in the main results section below (see Tables in Supplementary Materials for worthwhileness). The graph in Fig. 1 shows that overall SWB is very similar whether duration is accounted for or not. This holds for both measurement methods and both samples. This similarity also holds at an individual level, as evidenced by Fig. 2. To confirm this similarity, we conducted t-tests comparing the mean difference between intensity only SWB with duration-weighted SWB across methods and samples (see Table 2). We considered values above a 1% difference in SWB (0.1) to be a meaningful difference. Other papers have reported a difference of around 5% for major life events such as unemployment and disability, and so 1% makes it more likely that differences will be found^16^. The results show that overall SWB does not change by more than 1% whether duration is weighted or not. This result holds even when different methods of SWB are employed, such as when using daily averages of SWB reports vs. total averages of SWB over the course of the 2–3 week study (see Statistical Analysis in Supplementary Materials for specific calculations).

**Table 2:** Mean of pairwise differences between average reported SWB (happiness intensity × duration) with duration weights and average happiness without duration weights, with confidence interval at 95% level.

Mean difference	Student sample				Mixed sample			
	EMA		DRM		EMA		DRM	
	Total averages	Daily averages	Total averages	Daily averages	Total averages	Daily averages	Total averages	Daily averages
Happiness (CI@95%)	0.035*** [0.005, 0.064]	0.028*** [0.014, 0.042]	− 0.007*** [− 0.038, 0.023]	0.004*** [− 0.012, 0.020]	− 0.008*** [− 0.041, 0.025]	0.005*** [− 0.009, 0.018]	− 0.021*** [− 0.050, 0.008]	0.005*** [− 0.014, 0.023]

Significant results mean that mean difference is less than 1%. (***if *p* val < 0.001;**if *p* val < 0.01) (see Supplementary Materials, Table Table S13 for worthwhileness).

We conducted post-hoc analyses to explore several factors that might explain why weighting by duration might not affect overall SWB. First, we explored variation in the two core variables, intensity, and duration. We found that for individuals with daily activity patterns that result in higher variance in duration (see Supplementary Materials, Table S1), and/or in high variance in intensity reports (see Supplementary Materials, Table S2), the differences between duration-weighted and unweighted scores became greater, such that all the t-tests with respect to the total averages yielded results that were not statistically significant and thus greater than 1%.

Second, we explored the relationship between the two core variables: intensity and duration. For each person, we sub-sampled reports by selecting those that had a relatively higher correlation between intensity and duration. These reports were obtained by randomly sampling 100 sets containing half of each person’s reports and then picking the set with the highest correlation between intensity and duration. Interestingly, we found that duration did impact overall SWB for reports with these higher correlations: the higher the percentile of correlation (whether it was positive or negative) the greater the difference between weighted and non-weighted SWB (see Supplementary Materials Table S3). Moreover, the correlation was found to be the most important driver in explaining the difference between duration weighted and unweighted SWB, in contrast with the standard deviation of the SWB and duration variables (see Supplementary Materials, Tables S4 and S5 for details of the regression analysis). These three variables explain between 45 and 81% of the variance observed in the difference variable. We also note that it was not possible to increase the strength of the correlation between intensity and duration by focusing on either overall SWB reports, or SWB reports associated with specific activities, that were higher (happier) or lower (more miserable) (see Supplementary Materials, Tables S6, S7, S8, S9, S10 + S11, S12.

For robustness we also checked whether the difference between duration-weighted and unweighted SWB changed as a function of different demographic and interpersonal characteristics including high and low absolute happiness levels per person, personality, age, education, employment, gender, income. None of these variables explained the lack of relationship between duration and overall SWB. We also found no strong evidence for the possibility that happy activities make people happy up until a certain point as would be predicted by the law of diminishing marginal returns.

## Discussion

In an exploratory study, and in a follow up pre-registered experiment using diverse, multi-national samples, we found that weighting SWB reports by the duration of daily activities does not change overall SWB. This research challenges the assumption that the duration of emotional episodes makes a meaningful contribution to the calculation of SWB, calling into question the validity of current measurement tools. The result is robust to differences in SWB measurement, SWB calculation method (average of daily or total reports), high/low happiness experiences, as well as different demographic and interpersonal characteristics.

Our data highlight several possible explanations for this result. It appears that the low correlation between happiness intensity and duration reports is predominantly driving the result; reports with higher correlations (positive or negative) between intensity and duration yield larger differences in duration weighted and non-weighted SWB scores. More variation in scores typically generates stronger correlations. Indeed, in our data we found higher variance in intensity and/or duration reports to be associated with larger differences between duration and non-duration weighted happiness. Duration is (understandably) not expected to influence overall SWB if activities are always lasting roughly the same amount of time on average and/or if people report roughly the same happiness intensity on average.

These results call into question the reliability of existing happiness measures. Whilst it is possible that the low variation in duration and intensity (and therefore the lack of relationship between the overall SWB and duration) is a true reflection of how these entities vary in real life, this result is perhaps more likely to be a product of mismeasurement. In capturing duration associated with activities rather than emotional experience, it remains a possibility that current measures may fail to capture important variance in duration that is related to SWB. For example, although you may report feeling 4/10 happiness whilst commuting, that feeling might have been influenced by how you felt just before you started commuting. This “emotional lag” across activities means that the duration of this emotional episode may not be captured completely by focusing on commuting alone. Indeed, in our exploratory analysis, we show that happiness from the last emotional episode is a stronger predictor of current SWB reports than duration from the previous emotional episode. This lends support to the idea that the beginning and end of emotional experiences are not always clearly signalled by the beginning and end of any given activity—carryover effects are present (see Supplementary Materials, Table S14). Further manipulation studies where these two types of happiness measures (activity and emotion based) are directly compared will be necessary to affirm this possibility.

Moreover, associating duration with activities as a proxy for the duration of our emotional experiences may complicate the relationship between SWB intensity and its duration. Spending more time in an *activity* we like may start to yield less happiness after a while, whilst this is less likely to be the case for emotional experiences. A longer time spent feeling happy is unlikely to make us feel any less happy beyond a certain time since we are already capturing a direct measure of emotional experience. Importantly, previous research has identified a strong relationship between the duration of emotional states and their intensity when emotion is being measured directly in a study where participants were asked to provide daily reports on their experiences of anger, joy, or fear, and rate their intensity, higher emotional intensity was found to be significantly associated with longer durations for all three emotions^17^. Thus, a focus on activity duration instead of happiness duration may be obscuring the relationship between happiness duration and intensity.

## Conclusions

This research provides substance to the concerns of those already questioning the validity of self-reported happiness^12^ and should concern academics and practitioners that use SWB tools to evaluate impact. Providing that these findings are replicated in further studies, including those that directly compare activity and emotion based SWB reporting, new happiness measures may want to consider additional sources of measurement to complement, or perhaps even replace, self-report. For instance, identifying the exact start and end point of our emotional experiences will be a more cognitively demanding task than identifying the start and end point of activities. Therefore, affective computing approaches that generate an automatic mapping of additional variables predictive of certain emotional states for participants may yield more promising results in terms of capturing the full trajectory of emotional experience^18^. They will also be better able to detect other factors that may increase the variability of emotion duration such as emotional triggers^17^. For example, studies using smartwatches that detect heart beats and light exposure have found that happiness has an important association with these parameters^19^. Against this background, rapidly evolving new technologies that enable passive monitoring of these additional variables represent a promising avenue for more effective SWB monitoring tools in the future^20–22^.

In this study we were unable to discern the causal impact of differing reported happiness levels on the duration of activities and vice versa. This would require a manipulation study, where ecological validity would be reduced and exploration of the relationship between intensity and duration with respect to the most popular measurement tools would not be possible. Considering these findings this would be useful complementary research. Importantly, however, by focusing on EMA and DRM reports over the same period by the same people over time, our study design allowed for the isolation of differences in SWB at the episode level (how the same person reports emotion intensity and duration across measures in the same episode) and at the daily level (how the same person reports emotion intensity across measures on the same day). These interpersonal comparisons are critical for generating more robust and reliable approximations of SWB^23^. Despite the diversity of samples used in this study, we recommend that replications of this study with both similar and additional sub-samples (such as those with mental health problems) could be conducted to further improve generalizability of these results.

Going beyond previous work focused on how the frequency of positive experiences contribute to overall SWB, we show that duration does not meaningfully contribute to the calculation of SWB using existing measures. Our results do not rule out the fact that longer time spent in happy experiences is good for overall SWB. However, they do cast doubt on the ability of existing wellbeing measures to show that this is true. The reasons for this must take on an important new line of scientific research and perhaps new happiness measurements.

## Methods

### Participants

Phase 1 of the data generation was conducted in Spain, Colombia, Chile, Peru and UK (mixed sample). This sample were recruited between the dates 25/05/2018 and 24/09/2018 through a recruitment agency who attempted to diversify the sample with respect to age, gender, education, and socio-economic status. Phase 2 was conducted in the UK (student sample) between the dates of 29/12/2018 and 03/03/2019. The student sample were from the London School of Economics University in England and were recruited via authorised university communication channels including social media, university newsletters and email. The demographics for each sample can be found in Table 3 below. All participants signed a consent and privacy policy form on entering the study. Participants were only allowed to complete the study if they were: over 18, able to use a smartphone, and had no current mental health diagnosis (to control for the potential impact of mental health related medications). Only Android and iPhones were allowed in the study. However, since together these brands account for 99% of the global market share and this figure will be higher in the countries specified, we consider this to be a good representation of the population.

**Table 3:** Descriptives of study samples.

	Student sample	Mixed sample
Number of participants	217	195
Mean age	22.9 ± 3.7	31.5 ± 6.1
Mean income	£665 ± 555	€1964 ± 1073
Gender split	62% female, 38% male	58% female, 42% male
Employment	20% employed, 80% not employed	51% employed, 49% not employed
Life satisfaction	6.82 ± 1.66	7.16 ± 1.55

In this analysis, we started from a sample of 582 in the mixed group and 653 in the student group who had completed mood reports since this was the focus of our paper. From this sample, we excluded SWB reports where duration lasted longer than 12 h, as well as EMA reports where there appeared to be duplicates (two episodes reported within less than 15 min of each other). We included in the analysis the participants who submitted more than 20 EMA reports and at least 5 DRM reports in total, which implies that they were active for at least 5 days of the study. These criteria resulted in the exclusion of 387 participants from the mixed sample and 436 participants from the student sample. The final sample numbers are listed in the Table 3 below.

### Procedure

As part of a larger study exploring the determinants of SWB participants were instructed to download a custom designed mobile app via a specially curated study webpage. Once downloaded, participants underwent a series of initial onboarding questions via the app including questions relating to overall life satisfaction, worthwhileness, daily happiness and anxiety, and overall happiness and anxiety. These questions were followed by demographics and trait-based questionnaires including personality (50 item Big Five personality assessment questionnaire available on the IPIP website^24^). Participants then entered a SWB monitoring period of 2–3 weeks within the app during which they completed five daily EMA and once daily DRM reports, alongside several other SWB measures. The app instructed them re. When and how often to respond. Given the high intensity of SWB reports required, we considered this time frame as being sufficiently long enough to show change but not too long that too many of the sample would be lost. At the end of the study respondents were asked to complete the same initial questions relating to overall life satisfaction, worthwhileness, daily happiness and anxiety, and overall happiness and anxiety, that they received in the onboarding section.

Participants were able to report bug-related concerns via the app anonymously. These were responded to in app and anonymously by the assigned Alpha employees. Respondents with over 70% completion rate were reimbursed with £20 Amazon vouchers (student sample) and £40 (mixed sample). We decided that these incentives would be large enough to recruit a big enough *n* in the respective samples, but not sufficiently high enough to change people’s wellbeing. The higher price in the mixed sample was due to rates set by the recruitment agency used and reflective of a mostly working non-student sample.

### Measures

#### SWB measured by Ecological Momentary Assessment

EMA reports consisted of responses to various prompts issued at five random intervals throughout the day. First, participants had to select an activity (e.g. “working”) from a list of common activities in response to the prompt: “During the past hour, I was”. Activity lists differed depending on whether the sample was student or mixed. The student sample received common activities (e.g. “eating”) in addition to activities that were tailored to university life (e.g. “studying”). The mixed sample only received common activities. Next participants had to indicate the duration of the activity so far using a drop-down tab which showed time periods that went up in 10-min increments, ranging from 10 min to 4 h and 10 min, in response to the prompt: “How long have you been doing this?”. Then participants had to indicate who they were with, what they were thinking about, and where they were, from a list of common suggestions (e.g. “Kids”, “Events from my past”, “At my parents’ house”) in response to prompts: “I was with”, “I was thinking about”, “Where are you?”. Finally, participants had to answer how they felt on a scale of 0–10 in response to prompts: “How happy did you feel?” and “How worthwhile did this feel?”.

#### SWB measured by Day Reconstruction Method

Every morning, participants were asked to provide an overview of the previous day partitioned in episodes. We used the text from the DRM instructions provided in Kahneman et al.^4^: “Think of your day as a continuous series of scenes or episodes in a film. Give each episode a brief name that will help you remember it (for example, ‘commuting to work’ or ‘at lunch with B’…). Write down the approximate times at which each episode began and ended.”

Participants then had to indicate what they were doing (e.g. “working”) in response to the prompt “I was doing” and select a “start time” and “end time” for the episode. Activity lists differed depending on whether the sample was student or mixed, as described above in EMA reports. Also, like EMA -reports, for each episode participants had to indicate who they were with and what they were thinking about in response to prompts: “I was with” and “I was thinking about”. Finally, as per EMA reports, participants had to answer how they felt on a scale of 0–10 in response to prompts: “How happy did you feel?” and “How worthwhile did this feel?” Participants could not complete the DRM without having covered 12 h of emotional episodes.

#### SWB calculations

For robustness, we used four different formulas for calculating SWB: (1) total average SWB scores aggregated over the full length of the 2–3 week studies (Total SWB), (2) total average SWB scores aggregated over the full length of the 2–3 week studies weighted by duration (Total SWB weighted), (3) average of daily SWB scores (Daily SWB), (4) average of daily SWB scores weighted by duration (Daily SWB weighted). See below for details.

- **Total SWB** = $\frac{{SWB_{1} + SWB_{2} + \cdots + SWB_{Q} }}{Q}$, where SWB_i_ is the reported wellbeing associated to the i-th activity and Q is the number of questionnaires answered;
- **Total weighted SWB** = $\frac{{Dur_{1} SWB_{1} + Dur_{2} SWB_{2} + \cdots + Dur_{Q} SWB_{Q} }}{{Dur_{1} + Dur_{2} + \cdots Dur_{Q} }}$, where Dur_i_ is the reported duration of the i-th activity and SWB_i_ and Q are as above;
- **Daily SWB** = $\frac{{\frac{{(SWB_{1,1} + \cdots + SWB_{{Q_{1,1} }} )}}{{Q_{1} }} + \cdots + \frac{{(SWB_{1,D} + \cdots + SWB_{QD,D} )}}{{Q_{1} }}}}{D}$, where SWB_j,i_ is the reported wellbeing associated to the i-th activity on the j-th day, Qj is the number of questionnaires answered on the j-th day and D is the number of days the study lasted;
- **Daily weighted SWB** = $\frac{{\frac{{Dur_{1,1} SWB_{1,1} + \cdots + Dur_{{Q_{1,1} }} SWB_{{Q_{1,1} }} }}{{Dur_{1,1} + \cdots + Dur_{{Q_{1,1} }} }} + \cdots + \frac{{Dur_{1,D} SWB_{1,D} + \cdots + Dur_{QD,D} SWB_{QD,D} }}{{Dur_{1,D} + \cdots + Dur_{QD,D} }}}}{D}$, where Dur_i,j_ is the reported duration of the i-th activity on the j-th day and SWB_i,j_, Qj and D are as in the previous point.

## Supplementary Information

Supplementary Information.

## Data Availability

The data that support the findings of this study are available from Koa Health but restrictions apply to the availability of these data, which were used under license for the current study, and so are not publicly available. Data are however available from the authors upon reasonable request (with non-commercial intent) and with permission of Koa Health.
