# Supplementary material for: The duration of daily activities has no impact on measures of overall wellbeing
Source: Sci Rep. 2022 Jan 11;12:514. doi: 10.1038/s41598-021-04606-9 (PMC8752733; doi:10.1038/s41598-021-04606-9)
Supplement: Supplementary file 1 — Supplementary Information. [file 41598_2021_4606_MOESM1_ESM.docx]

Supplementary Materials for

**The duration of daily activities has no impact on measures of overall wellbeing**

**This PDF file includes:**

Materials (more detail)

Statistical Analysis (more detail)

Tables S1-S14

**Table of Contents**

1. Materials 3-4

1. Statistical Analysis 5
2. Tables S1-S14 6-19

### Materials

### Ecological Momentary Assessment


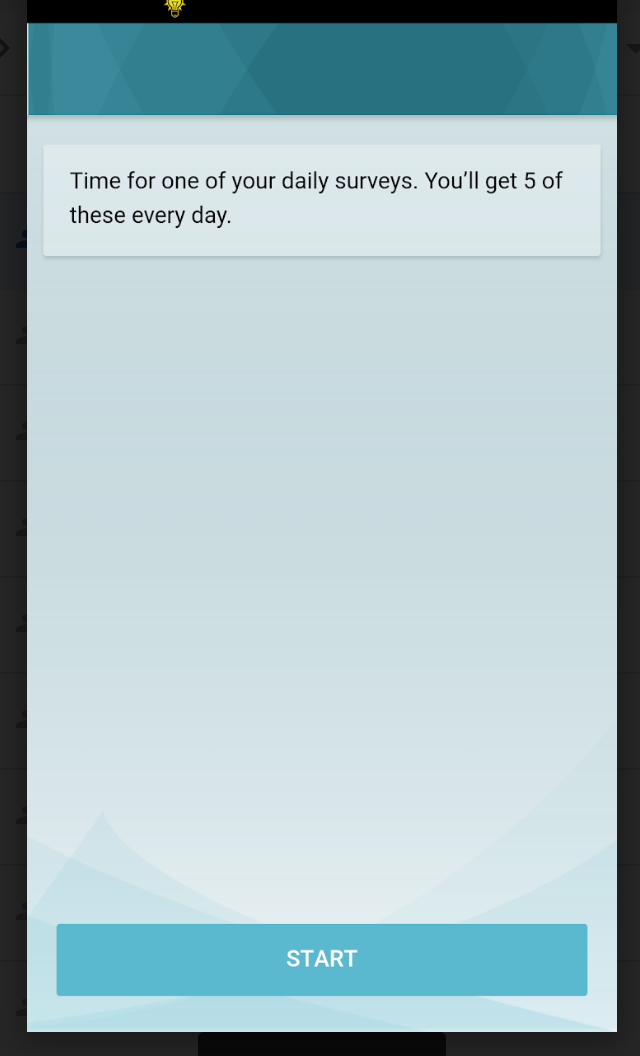

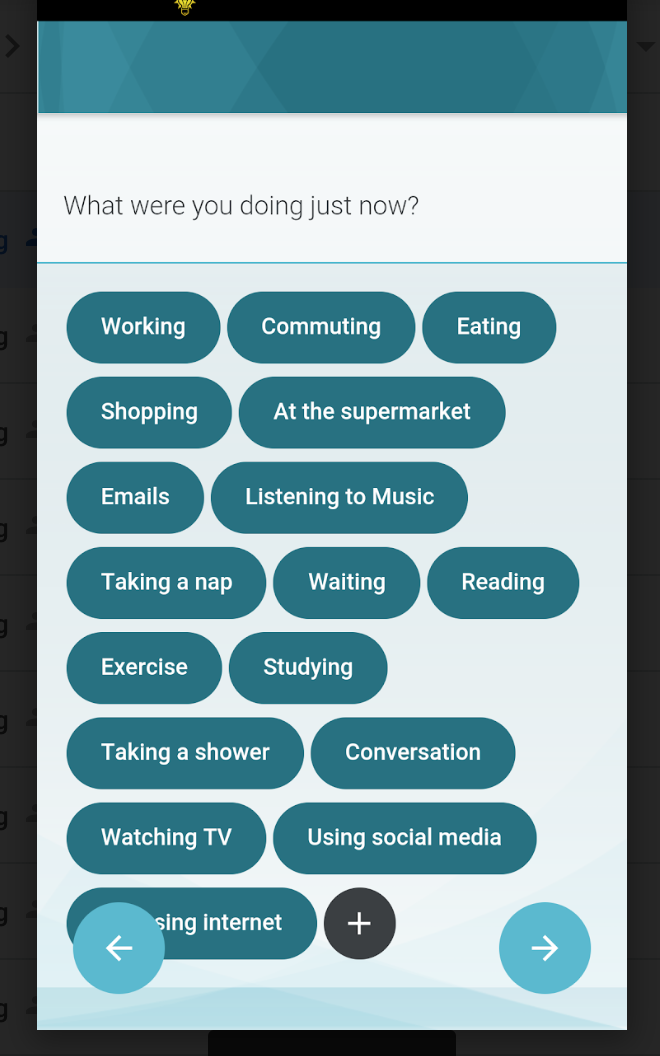

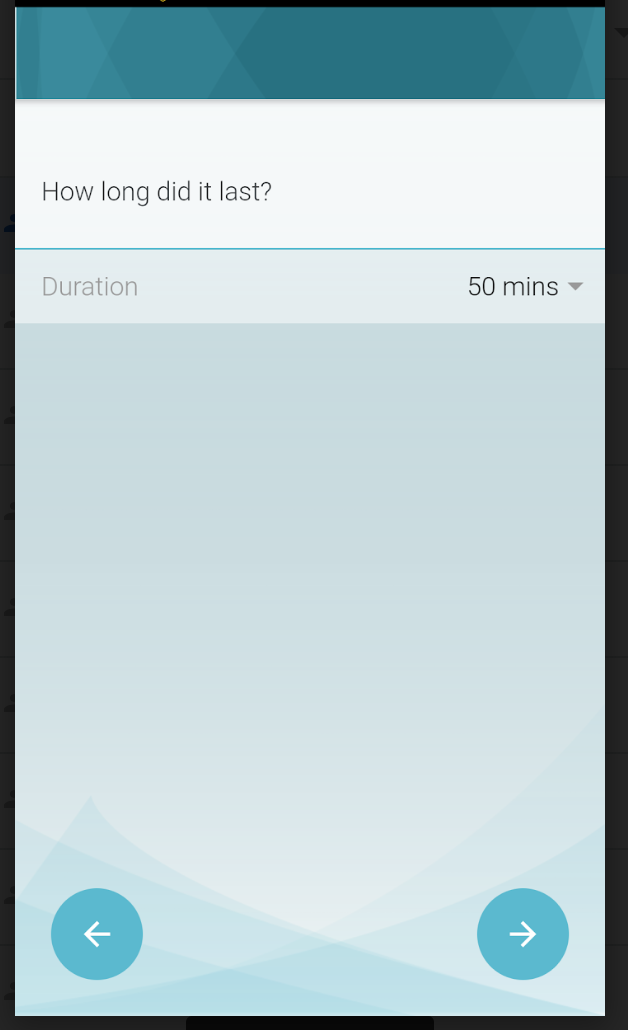


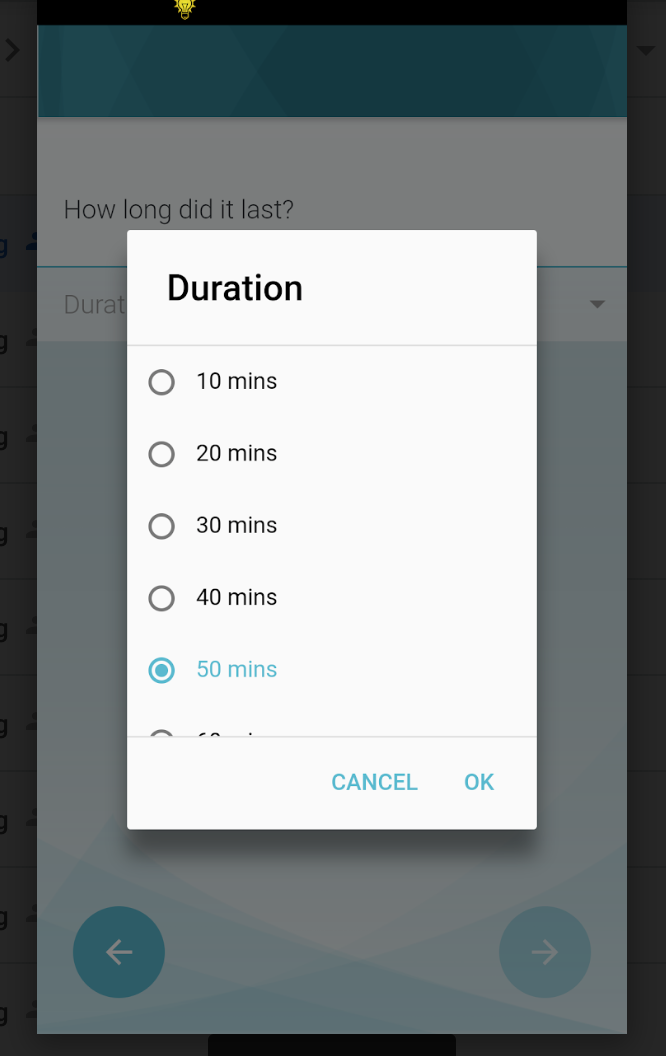

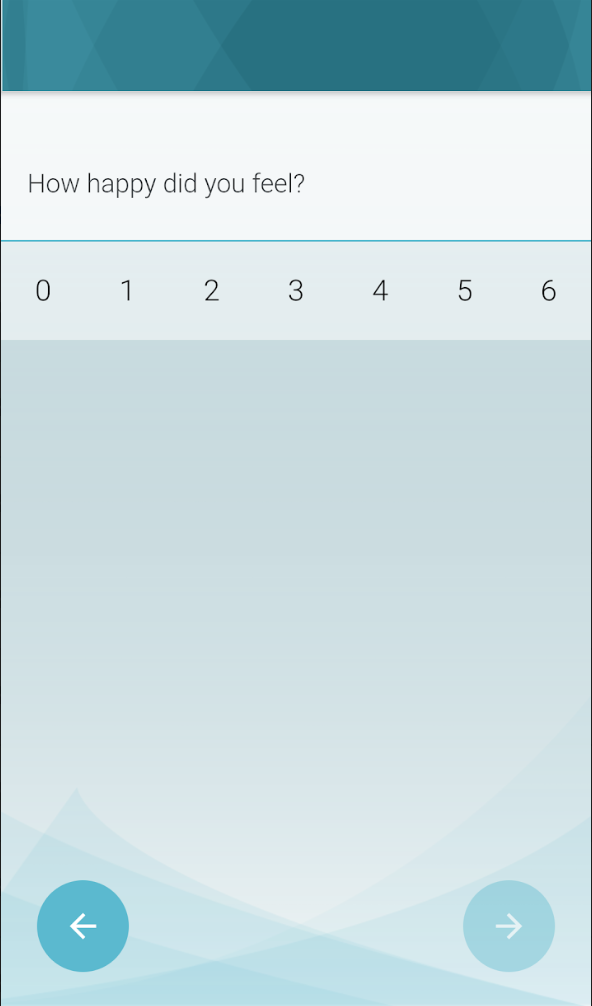

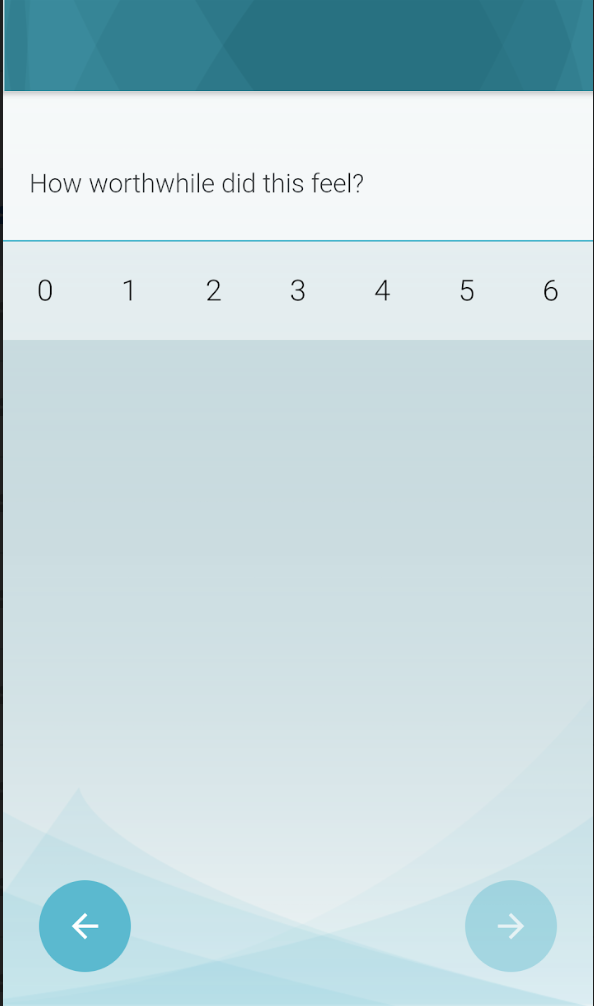


Screenshots of the Ecological Momentary Assessment measure taken from the Reflections app that was used to collect our data. Please note that the scale was changed from 0-6 to 0-10 before the app launch, however, these screenshots were taken just before that point and so we do not have the adjusted screens to display.

### Day Reconstruction Method


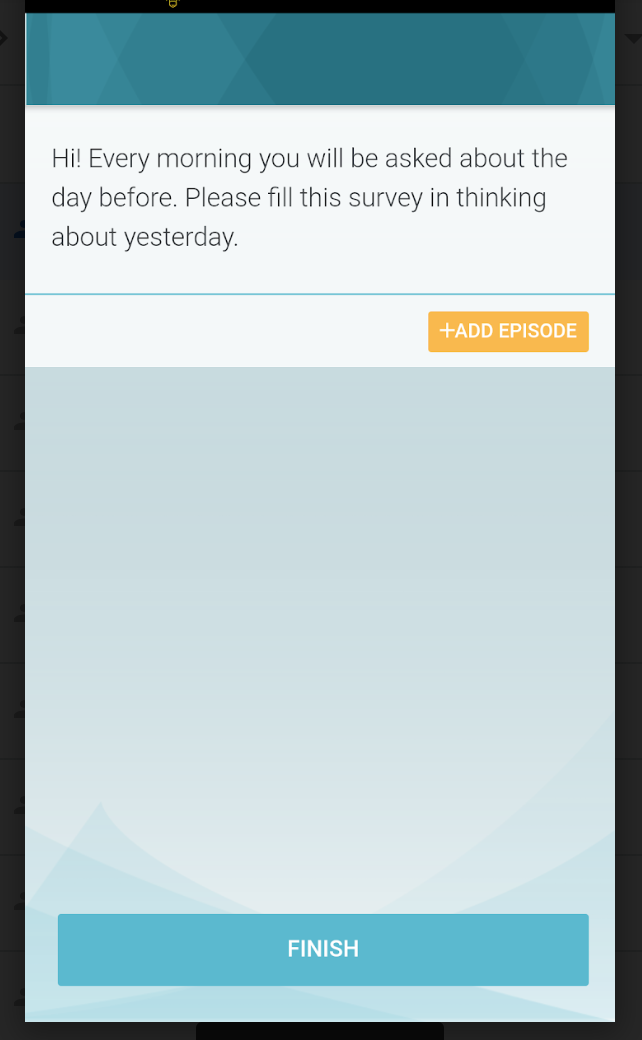

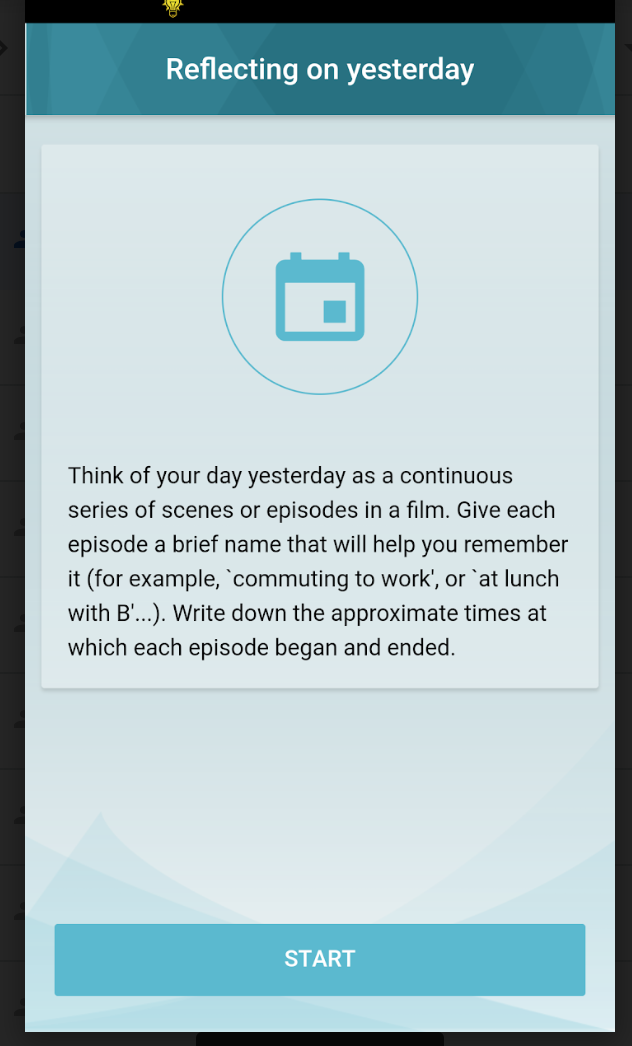

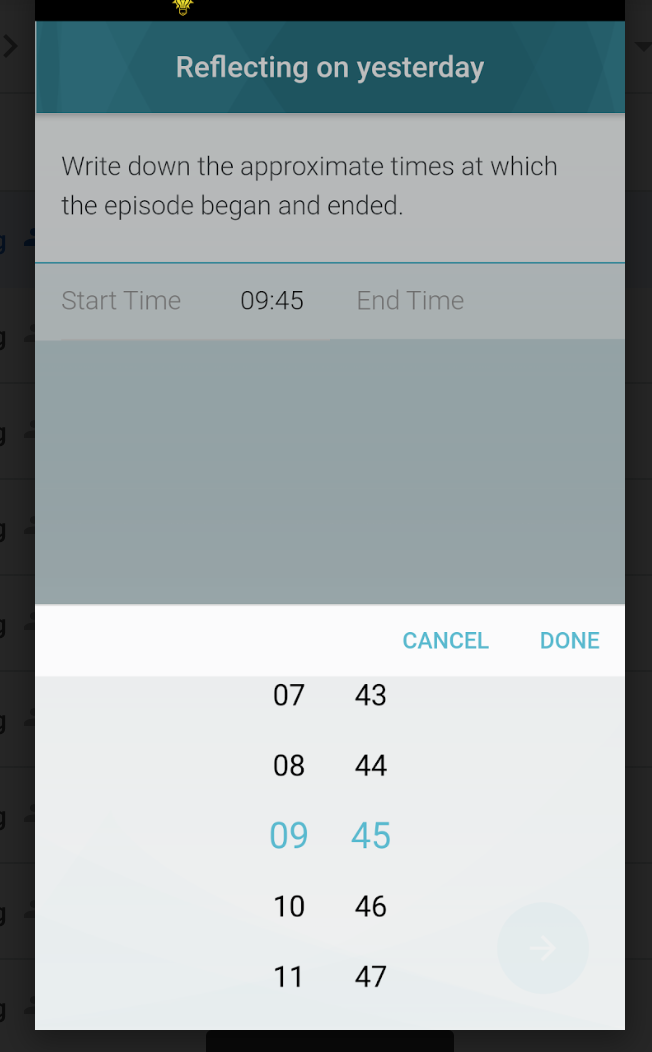


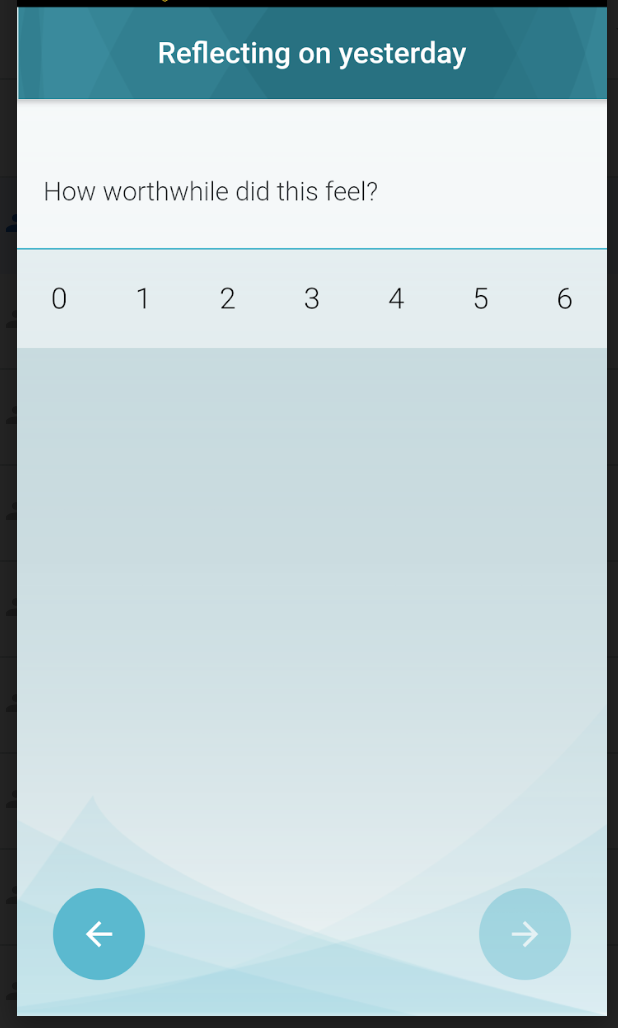

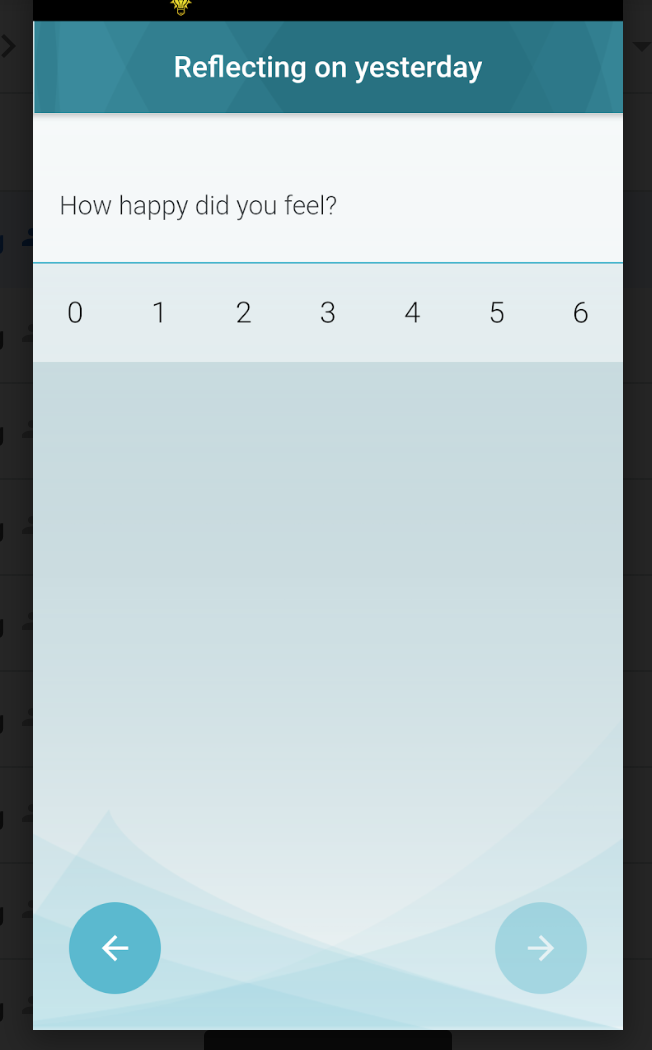


Screenshots of the Day Reconstruction Method measure taken from the Reflections app that was used to collect our data

1. **Statistical Analysis**

Robustness check for t-tests

For robustness, we used four different formulas for calculating SWB: 1) total average SWB scores aggregated over the full length of the 2-3 week studies (Total SWB), 2) total average SWB scores aggregated over the full length of the 2-3 week studies weighted by duration (Total SWB weighted), 3) average of daily SWB scores (Daily SWB), 4) average of daily SWB scores weighted by duration (Daily SWB weighted). See below for details.

- **Total SWB** =
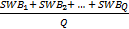

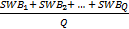
, where SWB_i_ is the reported wellbeing associated to the i-th activity and Q is the number of questionnaires answered;
- **Total *weighted* SWB** =
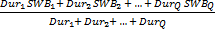

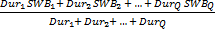
, where Dur_i_ is the reported duration of the i-th activity and SWB_i_ and Q are as above;
- **Daily SWB** =
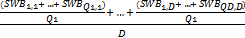

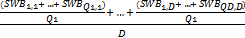
, where SWB_j,i_ is the reported wellbeing associated to the i-th activity on the j-th day, Qj is the number of questionnaires answered on the j-th day and D is the number of days the study lasted;
- **Daily *weighted* SWB** =
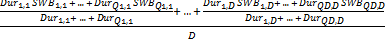

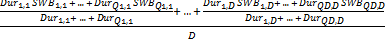
, where Dur_i,j_ is the reported duration of the i-th activity on the j-th day and SWB_i,j_, Qj and D are as in the previous point.

1. **Tables**

**Table S.1. High variance users - duration**

Mean of pairwise differences between average SWB with duration weights and average SWB without duration weights for each type of formula, SWB measure, questionnaire type, and sample. In computing Table S.1, we considered only those users who were in the top quartile of variance of duration of reports. Significant results mean that mean difference is less than a threshold of a tenth of a point. (*** if p-val < 0.001, ** if p-val < 0.01, * if p-val < 0.05).

| **Mean difference** | **Student sample** | | | | **Mixed sample** | | | |
| --- | --- | --- | --- | --- | --- | --- | --- | --- |
|  | **EMA** | | **DRM** | | **EMA** | | **DRM** | |
|  | **Total** | **Daily** | **Total** | **Daily** | **Total** | **Daily** | **Total** | **Daily** |
| **Happiness** | 0.059 | 0.039^***^ | -0.023^*^ | 0.003^***^ | 0.043 | 0.011^***^ | -0.077 | -0.024^***^ |
| **Worthwhileness** | 0.107 | 0.053^***^ | -0.008^**^ | 0.011^***^ | 0.077 | 0.039^***^ | -0.035 | -0.029^***^ |

**Table S.2. High variance users - intensity**

Mean of pairwise differences between average SWB with duration weights and average SWB without duration weights for each type of formula, SWB measure, questionnaire type, and sample. For Table S.2, we considered those who were in the top quartile of variance of SWB. Significant results mean that mean difference is less than a threshold of a tenth of a point. (*** if p-val < 0.001, ** if p-val < 0.01, * if p-val < 0.05).

| **Mean difference** | **Student sample** | | | | **Mixed sample** | | | |
| --- | --- | --- | --- | --- | --- | --- | --- | --- |
|  | **EMA** | | **DRM** | | **EMA** | | **DRM** | |
|  | **Total** | **Daily** | **Total** | **Daily** | **Total** | **Daily** | **Total** | **Daily** |
| **Happiness** | 0.083 | 0.064 | 0.042 | 0.016^***^ | -0.061 | -0.011^***^ | -0.073 | -0.038^*^ |
| **Worthwhileness** | 0.223 | 0.115 | 0.029 | 0.035^**^ | 0.044 | 0.013^**^ | 0.002 | -0.020^*^ |

**Table S.3. Simulations**. **Mean of pairwise differences between average happiness with duration weights and average happiness without duration weights.**

For each simulation, we randomly selected half of each users’ duration and intensity reports. In the average simulation, a single random sample is taken per user. In the low and high correlation simulations, 100 samples were taken per user and the ones with the lowest and highest correlation between happiness and duration were selected. Significant results mean that mean difference is less than a threshold of a tenth of a point. (*** if p-val < 0.001, ** if p-val < 0.01)

| **Mean difference** | **Student sample** | | **Mixed sample** | |
| --- | --- | --- | --- | --- |
|  | **EMA** | **DRM** | **EMA** | **DRM** |
| **Raw data** | 0.035*** | -0.007*** | -0.008*** | -0.021*** |
| **Average simulation** | 0.039** | -0.020*** | -0.023*** | -0.022*** |
| **Low correlation simulation** | 0.005*** | -0.012*** | -0.015*** | -0.005*** |
| **High correlation simulation** | 0.144 | 0.173 | 0.145 | 0.145 |

**Table S.4. Regression analysis. Total SWB.**

Estimation of the difference between average happiness with duration weights and average happiness without duration weights using as covariates the within-person correlation between happiness and duration reports, the standard deviation of happiness reports, and the standard deviation of duration reports. Data from EMA reports and the average happiness is computed using the Total formula.

|  | **Student sample**  $R^{2}=0.831$ | | | **Mixed sample**  $R^{2}=0.818$ | | |
| --- | --- | --- | --- | --- | --- | --- |
|  | **Coefficient** | **Std error** | **p-value** | **Coefficient** | **Std error** | **p-value** |
| **Constant** | -0.097 | 0.024 | <0.001 | -0.026 | 0.029 | 0.372 |
| **Correlation SWB/duration** | 0.913 | 0.028 | <0.001 | 1.075 | 0.038 | <0.001 |
| **Standard deviation SWB** | 0.052 | 0.014 | <0.001 | -0.013 | 0.013 | 0.346 |
| **Standard deviation duration** | <0.001 | <0.001 | 0.311 | <0.001 | <0.001 | 0.101 |

**Table S.5. Regression analysis. Daily SWB.**

Estimation of the difference between average happiness with duration weights and average happiness without duration weights using as covariates the within-person correlation between happiness and duration reports, the standard deviation of happiness reports, and the standard deviation of duration reports. Data from EMA reports and the average happiness is computed using the Daily formula.

|  | **Student sample**  $R^{2}=0.458$ | | | **Mixed sample**  $R^{2}=0.482$ | | |
| --- | --- | --- | --- | --- | --- | --- |
|  | **Coefficient** | **Std error** | **p-value** | **Coefficient** | **Std error** | **p-value** |
| **Constant** | -0.056 | 0.020 | 0.006 | 0.004 | 0.020 | 0.840 |
| **Correlation SWB/duration** | 0.315 | 0.024 | <0.001 | 0.329 | 0.026 | <0.001 |
| **Standard deviation SWB** | 0.027 | 0.012 | 0.021 | -0.004 | 0.009 | 0.690 |
| **Standard deviation duration** | <0.001 | <0.001 | 0.023 | <0.001 | <0.001 | 0.651 |

Low correlations between intensity and duration remained when grouping overall SWB reports by high or low happiness and when grouping activities with above or below average SWB.

**Table S.6. Correlation between SWB and duration by valence.**

Significant results marked *** if p-val < 0.001, ** if p-val < 0.01, * if p-val <0.05.

| **Correlation** | **Student sample** | | | | **Mixed sample** | | | |
| --- | --- | --- | --- | --- | --- | --- | --- | --- |
|  | **Happiness** | | **Worthwhileness** | | **Happiness** | | **Worthwhileness** | |
|  | **EMA** | **DRM** | **EMA** | **DRM** | **EMA** | **DRM** | **EMA** | **DRM** |
| **All episodes** | 0.01 | 0.02 | 0.06*** | 0.02 | -0.01 | 0.02* | 0.01 | 0.03*** |
| **High valence (>7)** | 0.07*** | 0.05* | 0.08*** | -0.01 | -0.02 | 0.01 | -0.01 | 0.01 |
| **Low valence (<5)** | -0.16*** | 0.03 | -0.10*** | 0.04 | -0.03 | 0.09* | -0.09** | -0.05 |

**Table S.7. Correlation between SWB and duration for activities with high mean SWB.** Significant results marked *** if p-val < 0.001, ** if p-val < 0.01, * if p-val <0.05.

| **Correlation** | **Student sample** | | | | **Mixed sample** | | | |
| --- | --- | --- | --- | --- | --- | --- | --- | --- |
|  | **Happiness** | | **Worthwhileness** | | **Happiness** | | **Worthwhileness** | |
|  | **EMA** | **DRM** | **EMA** | **DRM** | **EMA** | **DRM** | **EMA** | **DRM** |
| **Exercise** | 0.08 | 0.02 | -0.04 | -0.17** | 0.05 | 0.15* | 0.01 | -0.07 |
| **Conversation** | 0.19** | 0.09 | 0.14* | 0.01 | 0.17*** | -0.07 | 0.15*** | -0.12* |
| **Listening to music** | -0.01 | -0.10 | 0.20* | -0.03 | 0.12** | 0.03 | 0.08 | -0.04 |
| **Socialising** | 0.06 | 0.02 | 0.03 | 0.01 | NA | NA | NA | NA |

**Table S.8. Mean SWB for activities with high mean SWB.**

| **Mean SWB** | **Student sample** | | | | **Mixed sample** | | | |
| --- | --- | --- | --- | --- | --- | --- | --- | --- |
|  | **Happiness** | | **Worthwhileness** | | **Happiness** | | **Worthwhileness** | |
|  | **EMA** | **DRM** | **EMA** | **DRM** | **EMA** | **DRM** | **EMA** | **DRM** |
| **Exercise** | 7.01 | 7.21 | 7.05 | 7.40 | 7.94 | 7.74 | 8.16 | 8.17 |
| **Conversation** | 7.03 | 7.21 | 6.94 | 7.20 | 7.62 | 7.69 | 7.94 | 8.00 |
| **Listening to music** | 7.03 | 7.17 | 6.92 | 7.09 | 7.23 | 7.46 | 7.38 | 7.58 |
| **Socialising** | 7.18 | 7.50 | 6.81 | 7.02 | NA | NA | NA | NA |

**Table S.9. Correlation between SWB and duration for activities with low mean SWB.**

Significant results marked *** if p-val < 0.001, ** if p-val < 0.01, * if p-val <0.05.

| **Correlation** | **Student sample** | | | | **Mixed sample** | | | |
| --- | --- | --- | --- | --- | --- | --- | --- | --- |
|  | **Happiness** | | **Worthwhileness** | | **Happiness** | | **Worthwhileness** | |
|  | **EMA** | **DRM** | **EMA** | **DRM** | **EMA** | **DRM** | **EMA** | **DRM** |
| **Studying** | -0.01 | 0.04 | <0.01 | -0.04 | 0.07 | 0.04 | 0.07* | 0.02 |
| **Waiting** | -0.14 | 0.03 | 0.09 | 0.09 | -0.07 | -0.01 | -0.01 | -0.14 |
| **Commuting** | 0.02 | 0.04 | 0.07* | 0.03 | 0.02 | 0.13** | 0.06 | 0.13** |

**Table S.10. Mean SWB for activities with low mean SWB.**

| **Mean SWB** | **Student sample** | | | | **Mixed sample** | | | |
| --- | --- | --- | --- | --- | --- | --- | --- | --- |
|  | **Happiness** | | **Worthwhileness** | | **Happiness** | | **Worthwhileness** | |
|  | **EMA** | **DRM** | **EMA** | **DRM** | **EMA** | **DRM** | **EMA** | **DRM** |
| **Studying** | 6.21 | 6.21 | 6.56 | 6.70 | 6.78 | 6.63 | 7.49 | 7.42 |
| **Waiting** | 6.13 | 6.20 | 5.34 | 5.74 | 6.86 | 6.56 | 7.06 | 6.77 |
| **Commuting** | 6.26 | 6.28 | 6.11 | 6.11 | 6.87 | 6.80 | 6.90 | 7.03 |

**Table S.11. High valence episodes.**

Mean of pairwise differences between average SWB with duration weights and average SWB without duration weights for each type of formula, SWB measure, questionnaire type, and sample. In computing Table S.11, we considered only those episodes with SWB greater than 7. Significant results mean that mean difference is less than a threshold of a tenth of a point. (*** if p-val < 0.001, ** if p-val < 0.01, * if p-val < 0.05).

| **Mean difference** | **Student sample** | | | | **Mixed sample** | | | |
| --- | --- | --- | --- | --- | --- | --- | --- | --- |
|  | **EMA** | | **DRM** | | **EMA** | | **DRM** | |
|  | **Total** | **Daily** | **Total** | **Daily** | **Total** | **Daily** | **Total** | **Daily** |
| **Happiness** | 0.014*** | 0.003*** | 0.038*** | 0.012*** | 0.019*** | 0.007*** | 0.027*** | 0.017*** |
| **Worthwhileness** | 0.050*** | 0.010*** | 0.011*** | 0.008*** | 0.026*** | 0.009*** | 0.034*** | 0.015*** |

**Table S.12. Low valence episodes.**

Mean of pairwise differences between average SWB with duration weights and average SWB without duration weights for each type of formula, SWB measure, questionnaire type, and sample. In computing Table S.12, we considered only those episodes with SWB less than 5. Significant results mean that mean difference is less than a threshold of a tenth of a point. (*** if p-val < 0.001, ** if p-val < 0.01, * if p-val < 0.05).

| **Mean difference** | **Student sample** | | | | **Mixed sample** | | | |
| --- | --- | --- | --- | --- | --- | --- | --- | --- |
|  | **EMA** | | **DRM** | | **EMA** | | **DRM** | |
|  | **Total** | **Daily** | **Total** | **Daily** | **Total** | **Daily** | **Total** | **Daily** |
| **Happiness** | -0.013*** | 0.003*** | -0.008*** | -0.004*** | -0.031* | 0.001*** | 0.005*** | 0.019*** |
| **Worthwhileness** | -0.010*** | 0.004*** | -0.009*** | -0.002*** | -0.052 | -0.008*** | 0.035** | -0.002*** |

**Table S.13.** **Mean of pairwise differences between average happiness with duration weights and average worthwhileness without duration weights.** Significant results mean that mean difference is less than 1%. (*** if p-val < 0.001, ** if p-val < 0.01).

| **Mean difference** | **Student sample** | | | | **Mixed sample** | | | |
| --- | --- | --- | --- | --- | --- | --- | --- | --- |
|  | **EMA** | | **DRM** | | **EMA** | | **DRM** | |
|  | **Total averages** | **Daily averages** | **Total averages** | **Daily averages** | **Total averages** | **Daily averages** | **Total averages** | **Daily averages** |
| **Worthwhileness** | 0.151 | 0.077^*^ | 0.017^***^ | 0.030^***^ | 0.046^**^ | 0.028^***^ | 0.011^***^ | 0.006^***^ |

**Table S.14.** **Regression analysis. Happiness v. duration as predictors of current wellbeing report.** We ran a regression analysis with current wellbeing (happiness intensity / its duration) as a dependent variable and compared the contribution of the following independent variables: duration report of the previous episode, happiness report of the previous episode. For each individual, we considered each activity that appears at least 3 times in individual’s reports. We only looked at previous reports if they occurred on the same day as the current report. Happiness was taken as the maximum score of from people’s happiness and worthwhileness reports. Significant results mean that mean difference is less than 1%. (*** if p-val < 0.001, ** if p-val < 0.01).

|  | **Coefficient** | **Standard error** | **p value** |
| --- | --- | --- | --- |
| **Constant** | 3.690 | 0.447 | 3.59e-16*** |
| **Happiness from last reported episode (same day)** | 0.527 | 0.062 | < 2e-16 *** |
| **Duration from last reported episode** | -0.659 | 0.084 | 8.98e-15*** |

This model shows that the duration of the current as well as the previous episode are strong predictors of the wellbeing variance reported per episode. R2 = 0.07,  i.e. this model explains 7% of the variance in the reported wellbeing.
